# Analysis of the clinical value of combined monitoring of methylation transferase Wilms' tumour 1-associating protein and lipoprotein-associated phospholipase A2 in patients with coronary artery disease

**DOI:** 10.3389/fcvm.2025.1540047

**Published:** 2025-12-04

**Authors:** Fengxia Guo, Bing Hu, Gang Li

**Affiliations:** 1 Department of Clinical Laboratory, Henan Provincial People’s Hospital, People’s Hospital of Zhengzhou University, Zhengzhou, Henan, China; 2 Department of Clinical Laboratory, Affiliated Cancer Hospital of Zhengzhou University, Zhengzhou, Henan, China

**Keywords:** coronary artery disease, WTAP, Lp-PLA2, inflammatory markers, methylation transferase

## Abstract

**Objective:**

Clinical significance and correlation study of methyltransferase Wilms' tumour 1-associated protein (WTAP) and lipoprotein-associated phospholipase A2 (Lp-PLA2) in patients with coronary artery disease (CAD).

**Methods:**

This study enrolled 282 symptomatic patients with suspected CAD. Serum biomarkers including lipids, inflammatory markers (CRP, Lp-PLA2), and WTAP mRNA levels (quantified via RT-PCR) were analyzed. Coronary severity was assessed using Gensini scores.

**Results:**

CAD patients exhibited significantly elevated WTAP expression and higher Lp-PLA2 levels. WTAP correlated strongly with Lp-PLA2, CRP, and Gensini scores. Multivariate analysis identified WTAP and Lp-PLA2 as independent CAD predictors. Combined WTAP/Lp-PLA2 detection demonstrated superior diagnostic performance (AUC = 0.9548, sensitivity = 95.26%, specificity = 90.16%).

**Conclusion:**

WTAP and Lp-PLA2 synergistically reflect CAD progression, offering dual biomarkers for risk stratification.

## Introduction

Emerging evidence indicates that inflammatory biomarkers play a pivotal role in CAD pathogenesis and may serve as potential therapeutic targets for disease prevention and management. Current diagnostic approaches predominantly rely on imaging modalities, including invasive techniques such as intravascular ultrasound (1) and optical coherence tomography (2), as well as non-invasive methods like CT scans (3), MRI (4), and PET (5). However, invasive procedures are associated with procedural risks, high costs, and limited suitability for population-wide screening or longitudinal disease monitoring. Conversely, non-invasive imaging lacks sufficient sensitivity and specificity to fulfill clinical demands. Consequently, the identification of novel biomarkers holds significant promise for early CAD detection and risk stratification.

Wilms' tumor 1-associated protein (WTAP), a critical component of m6A methyltransferase complexes (6). WTAP has been implicated in arterial restenosis through m6A-mediated epigenetic regulation (7). Notably, WTAP mRNA expression exhibits a positive correlation with pro-inflammatory cytokine levels (8). Lipoprotein-associated phospholipase A2 (Lp-PLA2), predominantly secreted by neutrophils and macrophages within atherosclerotic plaques and subsequently transported via cholesterol particles, serves as a vascular-specific inflammatory marker (9, 10). Accumulating studies (11–13) demonstrate that Lp-PLA2 exacerbates atherosclerosis by amplifying inflammatory cascades. These findings collectively highlight the potential of WTAP and Lp-PLA2 in modulating inflammatory pathways underlying cardiovascular pathologies. Nevertheless, the clinical utility of WTAP and Lp-PLA2 quantification in CAD remains underexplored. This study aims to investigate circulating levels of WTAP and Lp-PLA2 in CAD patients, elucidate their association with disease progression, and evaluate their translational relevance in clinical practice.

## Materials and methods

### Research subjects

**Figure 1:**
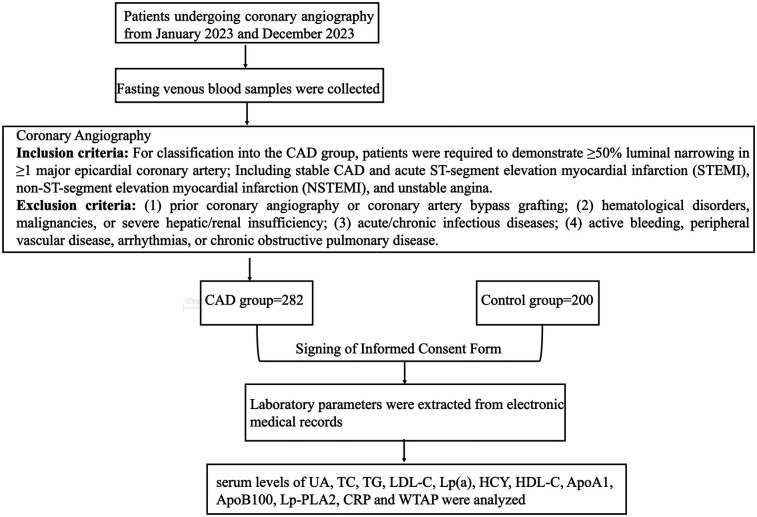
Flow chart.

This study enrolled 282 patients with CAD admitted to the Department of Cardiology at Henan Provincial People's Hospital between January 2023 and December 2023. Inclusion criteria required patients to meet established diagnostic criteria for CAD subtypes, including stable CAD and acute coronary syndrome [acute ST-segment elevation myocardial infarction (STEMI), non-ST-segment elevation myocardial infarction (NSTEMI), and unstable angina] (14, 15). For classification into the CAD group, patients were required to demonstrate ≥50% luminal narrowing in ≥1 major epicardial coronary artery, as this threshold is widely accepted to represent hemodynamically significant stenosis capable of impairing myocardial perfusion and contributing to clinical symptoms or ischemic events. Exclusion criteria included: (1) prior coronary angiography or coronary artery bypass grafting; (2) hematological disorders, malignancies, or severe hepatic/renal insufficiency; (3) acute/chronic infectious diseases; (4) active bleeding, peripheral vascular disease, arrhythmias, or chronic obstructive pulmonary disease. A control group of 200 age- and sex-matched healthy individuals undergoing routine medical examinations was concurrently enrolled. No statistically significant intergroup differences were observed in baseline characteristics (e.g., age, sex; *P* > 0.05). The study protocol was approved by the Ethics Committee of Henan Provincial People's Hospital (Approval No. 2023-42). All participants provided written informed consent after receiving detailed explanations of the study's purpose, procedures, blood sampling protocol, and coronary angiography requirements (Figure 1).

### Specimen collection and processing

Laboratory parameters were extracted from electronic medical records. Fasting venous blood samples (5 mL) were were obtained prior to coronary angiography to avoid potential bias from knowledge of coronary anatomy. After centrifugation at 3,000×*g* for 15 min at 4 °C, plasma levels of uric acid (UA), total cholesterol (TC), triglycerides (TG), low-density lipoprotein cholesterol (LDL-C), lipoprotein(a) [Lp(a)], homocysteine (HCY), high-density lipoprotein cholesterol (HDL-C), apolipoprotein A1 (ApoA1), apolipoprotein B100 (ApoB100), and lipoprotein-associated phospholipase A2 (Lp-PLA2) were quantified using an Abbott ARCHITECT c16000 automated biochemistry analyzer. C-reactive protein (CRP) levels were measured with a Purmo PA-990 specific protein analyzer. Residual specimens were aliquoted and stored at −80 °C for subsequent analyses.

### Quantitative real-time PCR (qRT-PCR)

Total RNA was isolated from cultured cells using TRIzol reagent (Invitrogen, Carlsbad, CA, USA) and reverse-transcribed into cDNA. qRT-PCR was performed on a LightCycler 480 II system (Roche, Pleasanton, USA) with SYBR Green detection (TaKaRa Bio, RR420A, USA). Primer sequences were as follows:

WTAP Forward: 5′-TTTCCACTCCCACCAGGAAAG-3′;
WTAP Reverse: 5′-TAAGACTGCCATCTGGACCG-3′.

GAPDH served as the endogenous control. Relative expression levels were calculated using the 2−^ΔΔ^Ct method. All reactions were conducted in triplicate.

### Gensini score

Coronary lesion severity was quantified using the Gensini scoring system. Segment-specific weighting factors (0.5–5.0) were multiplied by stenosis severity scores as follows: 32 for total occlusion (100%), 16 for 99% stenosis, 8 for 90%, 4 for 75%, 2 for 50%, and 1 for 25% lumen reduction. Scores were aggregated across four major coronary arteries (left main, left anterior descending, left circumflex, and right coronary artery) following standardized methodologies (16).

### Statistical analysis

Data were analyzed using SPSS version 24.0 (IBM Corp.). Normality was assessed via the Kolmogorov–Smirnov test. Normally distributed variables are presented as mean ± standard deviation (SD) and compared using independent *t*-tests (for two groups) or one-way ANOVA (for multiple groups). Nonparametric data are expressed as median (interquartile range) and analyzed with Mann–Whitney *U* or Kruskal–Wallis tests. Correlation analyses employed Pearson (normal data) or Spearman (non-normal data) coefficients. To identify independent correlates of CAD, variables with *P* < 0.1 in univariate analyses were included in a multivariate logistic regression model. Receiver operating characteristic (ROC) curves were generated to evaluate the diagnostic performance of WTAP. A two-tailed *P* < 0.05 was considered statistically significant.

## Results

### 1. Comparison of clinical characteristics between CAD and control groups

The study revealed no statistically significant differences (*P* > 0.05) in age, sex, TG, LDL-C, Lp(a), HCY, HDL-C, ApoA1, ApoB100, or UA levels between the CAD group and the control group. However, significant differences (*P* < 0.05) were observed in TC, CRP, and Lp-PLA2 levels (Table 1).

**Table 1:** Comparison of clinical characteristics.

Group	Control	CAD	*t*/*χ*^2^	*P*
Age (Year)	55.3 ± 10.02	56.03 ± 5.19	0.92	0.359
Sex (Male/Female)	98/102	119/163	1.13	0.054
Hypertension	90/110	120/142	10.7	0.301
Diabetes	86/114	136/146	1.276	0.089
Smoking	89/111	123/159	2.32	0.137
TG (mmol/L)	1.34 ± 0.12	1.39 ± 0.24	0.65	0.688
TC (mmol/L)	1.03 ± 0.79	4.1 ± 0.83	14.32	0.001
LDL-C (mmol/L)	2.88 ± 0.78	2.96 ± 0.84	0.49	0.603
Lp(a) (mg/dL)	129.45 ± 1.34	141.9 ± 2.19	0.64	0.087
HCY (umol/L)	10.2 ± 1.40	11.45 ± 2.43	0.83	0.059
HDL-C (mmol/L)	1.13 ± 0.47	1.05 ± 0.22	0.50	0.074
ApoA1 (g/L)	1.43 ± 0.38	1.45 ± 0.30	1.91	0.183
ApoB100 (g/L)	0.84 ± 0.29	0.87 ± 0.12	3.65	0.063
CRP (mg/dL)	5.90 ± 3.18	30.28 ± 6.70	13.24	<0.001
UA (umol/L)	302.4 ± 79.50	322 ± 67.50	1.31	0.311
Lp-PLA2 (U/L)	165.34 ± 21.75	723.29 ± 22.34	25.42	<0.001

### 2. WTAP expression analysis in CAD patients

**Figure 2:**
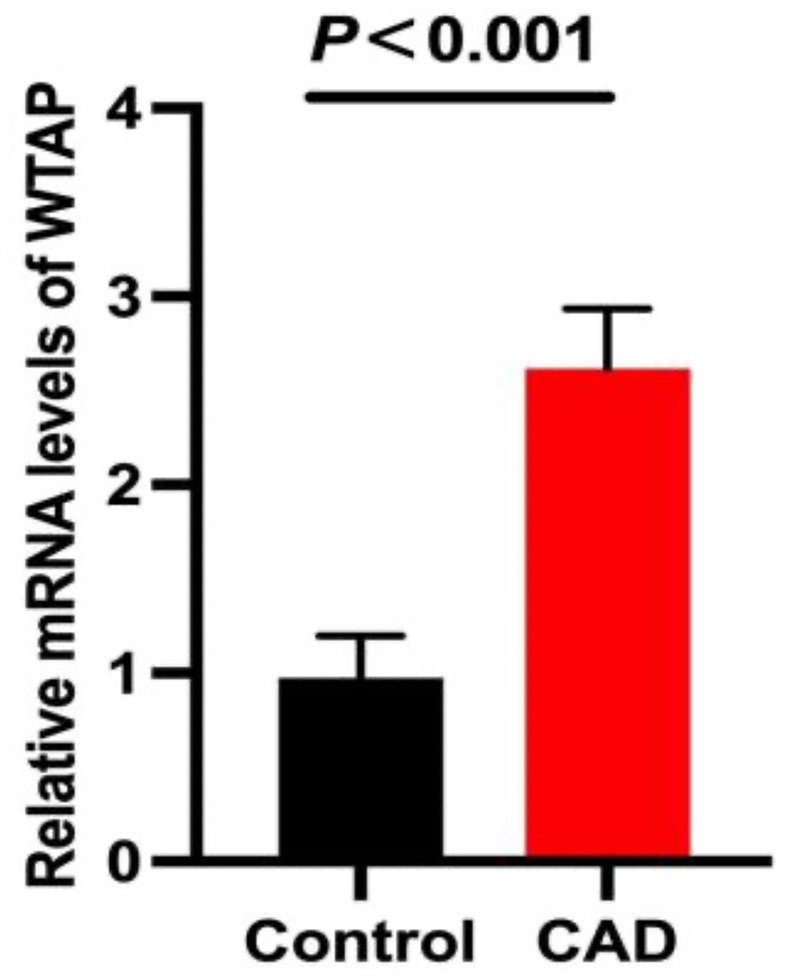
WTAP expression analysis in CAD patients.

WTAP expression levels were significantly upregulated in the CAD group relative to controls (*P* < 0.05) (Figure 2).

### 3. Correlation between Lp-PLA2 and inflammatory markers/gensini score

Lp-PLA2 showed significant positive correlations with CRP and the Gensini score in CAD patients (*P* < 0.05) (Table 2).

**Table 2:** Correlation between Lp-PLA2 and inflammatory markers/gensini score.

Lp-PLA2	TG	Lpa	HCY	HDL-C	ApoA1	ApoB100	TC
R	0.271	0.218	0.193	0.143	−0.292	0.123	0.382
*P*	0.064	0.175	0.204	0.169	0.403	0.32	0.078
Lp-PLA2	LDL-C	UA	Gensini	CRP			
R	0.641	0.193	0.493	0.54			
*P*	0.392	0.54	0.027	0.004			

R denotes the correlation coefficient.

### 4. Correlation between WTAP and inflammatory markers/gensini score

WTAP expression significantly correlated with CRP and the Gensini score in CAD patients (*P* < 0.05) (Table 3).

**Table 3:** Correlation between WTAP and inflammatory markers/gensini score.

WTAP	TG	Lpa	HCY	HDL-C	ApoA1	ApoB100	TC
R	0.18	0.23	0.09	0.05	0.32	0.12	0.42
*P*	0.12	0.22	0.36	0.17	0.06	0.10	0.06
WTAP	LDL-C	UA	Gensini	CRP			
R	0.08	0.21	0.54	0.47			
*P*	0.21	0.38	0.01	0.02			

### 5. Logistic regression analysis

Variables with *P* < 0.1 in the univariate analysis were entered into a multivariate logistic regression model. The multivariate analysis demonstrated that WTAP and Lp-PLA2 were independent risk factors for CAD (*P* < 0.001; Tables 4, 5).

**Table 4:** Univariate logistic regression analysis.

Parameters	*B*	SE	Wald	OR (95% CI)	*P*
TG	0.008	0.011	1.602	1.011 (0.984,1.247)	0.184
TC	0.002	0.134	2.350	1.653 (1.209,2.016)	0.007
Lp(a)	0.152	0.015	1.151	1.459 (1.120,1.895)	0.253
HCY	0.367	0.004	1.071	0.998 (0.847,1.121)	0.224
HDL-C	0.029	0.200	1.085	1.131 (1.063,1.942)	0.889
LDL-C	0.016	0.141	1.682	1.446 (1.009,2.093)	0.285
ApoA1	0.169	0.032	0.247	1.002 (0.994,1.789)	0.491
ApoB100	0.095	0.003	1.439	1.006 (0.904,1.529)	0.183
UA	0.291	0.018	1.835	0.992 (0.967,1.008)	0.286
CRP	0.018	0.091	0.994	1.006 (0.915,1.407)	0.006
WTAP	0.034	0.045	12.910	1.137 (1.004,1.794)	<0.001
Lp-PLA2	0.029	0.185	10.235	1.899 (1.456,2.139)	<0.001

**Table 5:** Multivariate logistic regression analysis.

Parameters	*B*	SE	Wald	OR (95% CI)	*P*
TC	0.019	0.021	1.876	0.992 (0.967,0.999)	0.587
CRP	0.016	0.040	1.840	1.008 (0.893,1.328)	0.760
WTAP	0.070	0.010	10.738	1.007 (1.004,1.015)	<0.001
Lp-PLA2	0.050	0.030	13.080	1.002 (1.001,1.029)	<0.001

### 6. ROC curve analysis for diagnostic performance

ROC curve analysis revealed that the combined detection of Lp-PLA2 and WTAP achieved optimal diagnostic performance for CAD, with a sensitivity of 95.26%, specificity of 90.16%, and AUC of 0.9548 (Table 6).

**Table 6:** ROC curve analysis for diagnostic performance.

Parameters	Sensitivity%	specificity %	Cut-off	AUC
WTAP	81.38	89.73	2.98	87.37
Lp-PLA2	78.27	69.27	745.64	82.18
WTAP + Lp-PLA2	95.26	90.16	—	95.48

## Disscussion

Coronary artery disease (CAD), recognized as the primary pathological basis for cardiovascular morbidity and the leading cause of global mortality, remains challenging to detect in its early stages despite its clinical significance. Pathophysiological investigations have established the pivotal role of inflammatory responses in CAD progression (17). While conventional inflammatory biomarkers such as the neutrophil-lymphocyte ratio (NLR), interleukin-8 (IL-8), interleukin-10 (IL-10), and high-sensitivity C-reactive protein (hs-CRP) demonstrate predictive value for CAD detection, their clinical utility is limited by environmental susceptibility and instability. This underscores the necessity to identify stable inflammatory biomarkers for early CAD diagnosis.

Emerging evidence highlights the regulatory role of N6-methyladenosine (m6A) modifications in inflammatory pathways. Specifically, WTAP modulates m6A modifications in THP-1 macrophages to mediate anti-inflammatory effects (18) and exacerbates myocardial ischemia/reperfusion injury via ATF4 mRNA methylation, thereby enhancing apoptosis and endoplasmic reticulum stress (19). Notably, Wu et al. observed significantly reduced m6A methylation levels in leukocytes from both ankylosing spondylitis patients and murine models (20). WTAP-mediated m6A modification of KLF6 was shown to aggravate hypoxia/reoxygenation-induced apoptosis, inflammatory responses, oxidative stress, and ferroptosis in human cardiomyocytes (21). Concurrently, lipoprotein-associated phospholipase A2 (Lp-PLA2) has emerged as a critical mediator in cardiovascular pathology. Elevated Lp-PLA2 levels correlate strongly with acute coronary syndromes (22, 23) and predict recurrent cardiovascular events, including angina pectoris, myocardial infarction, and heart failure. Mechanistically, Lp-PLA2 accelerates coronary stenosis and atherosclerotic plaque rupture through oxidized fatty acid metabolism and lysophosphatidylcholine production (24, 25). Despite these advances, the diagnostic implications of WTAP and Lp-PLA2 expression patterns in CAD progression remain underexplored.

Our study demonstrated significant elevations in serum WTAP and Lp-PLA2 levels among CAD patients, with positive correlations to inflammatory markers and Gensini scores. These findings position both biomarkers as independent risk factors for CAD severity. Notably, ROC analysis revealed superior diagnostic specificity for WTAP compared to Lp-PLA2, while combined detection enhanced both sensitivity and specificity. This dual-biomarker approach may substantially improve CAD risk stratification and early diagnosis. Incorporating additional biomarkers, such as oxidized LDL and inflammatory cytokines, could provide a more comprehensive risk assessment profile for CAD. Future studies should examine the combined predictive power of these biomarkers to improve early diagnosis and prognosis.

Nevertheless, this study has limitations. First, the single-center design and predominantly Han Chinese cohort (>95%) from Central China may restrict the generalizability of our findings to other ethnicities and regions. Second, confounding factors, including lifestyle variations and medication regimens, were not fully controlled. Future multicenter studies with expanded sample sizes should address these constraints. A longitudinal design tracking biomarker trajectories over time would strengthen predictive validity and clarify their utility in early detection and disease progression monitoring. Furthermore, the precise mechanisms by which WTAP-regulated m6A modifications influence endothelial function and Lp-PLA2 modulates inflammatory cascades in atherogenesis warrant further mechanistic investigation.

In conclusion, our findings establish WTAP and Lp-PLA2 as novel inflammatory biomarkers for CAD, independent of traditional risk factors. Combined assessment of these biomarkers enhances diagnostic accuracy, offering clinical potential for early intervention and improved prognostic strategies in CAD management.

## Data Availability

The raw data supporting the conclusions of this article will be made available by the authors, without undue reservation.
